# Comparison of Cryoballoon and Ablation Index-Guided Radiofrequency Ablation in Paroxysmal Atrial Fibrillation

**DOI:** 10.3390/jcm14062119

**Published:** 2025-03-20

**Authors:** Botond Bocz, Dorottya Debreceni, Kristof-Ferenc Jánosi, Dalma Torma, Peter Kupo

**Affiliations:** Heart Institute, Medical School, University of Pecs, 7624 Pecs, Hungary; bocz.botond2@pte.hu (B.B.); debreceni.dorottya@pte.hu (D.D.); janosi.kristof@pte.hu (K.-F.J.); torma.dalma@pte.hu (D.T.)

**Keywords:** atrial fibrillation, ablation index, cryoballoon ablation and pulmonary vein isolation

## Abstract

**Background**: Atrial fibrillation is the most common sustained arrhythmia worldwide. Pulmonary vein isolation (PVI) is the most effective catheter ablation technique for treating paroxysmal atrial fibrillation (pAF). Common ablation methods include point-by-point radiofrequency (RF) ablation and single-shot techniques such as cryoballoon ablation (CB). This single-center, prospective study aimed to compare the efficacy of ablation index-guided RF ablation (AI-RF) and CB in patients with symptomatic, antiarrhythmic-resistant pAF. **Methods**: A total of 154 patients undergoing initial PVI were divided into two groups (CB: 51, AI-RF: 103), based on the operators’ decision. Procedural data (total procedure time, fluoroscopy time, radiation dose, complication rate) and recurrence rates were analyzed over a 12-month follow-up period. **Results**: The CB group had a significantly shorter total procedure time compared to the AI-RF group (64 [57; 74.8] minutes vs. 92 [76; 119] minutes; *p* < 0.001). However, the CB group experienced higher fluoroscopy times (559 [395; 868] seconds vs. 167 [126; 224] seconds; *p* < 0.001) and a greater fluoroscopy dose (21.8 [11.7; 40.1] mGy vs. 7.65 [5.21; 14.5] mGy; *p* < 0.001). Recurrence rates were similar during both the blanking period (11.7% vs. 10.7%; *p* = 0.84) and the 12-month follow-up period (22.7% vs. 13.4%; *p* = 0.22). No major complications were reported during this study. **Conclusions**: In this single-center study, there were no significant differences in long-term recurrence or complication rates between the CB and AI-RF groups for patients with antiarrhythmic-refractory, symptomatic pAF. While the CB group benefited from a significantly shorter procedure time, it required a higher fluoroscopy dose and a longer fluoroscopy time.

## 1. Introduction

Atrial fibrillation (AF) is the most common sustained cardiac arrythmia worldwide, ranging from 2% to 4% in the adult population. Patients with AF have a significantly higher risk of cardiovascular and cerebrovascular diseases, such as heart failure, myocardial infarction, and ischemic stroke [1]. According to the latest ESC Guidelines for the diagnosis and management of AF, the main reason for employing a rhythm control strategy is to reduce symptoms associated with AF and enhance the overall quality of life [2,3].

Catheter ablation is more effective than antiarrhythmic drugs (AADs) in maintaining sinus rhythm for AF. The electrical isolation of the pulmonary veins (PVs) represents the cornerstones of AF ablation procedures. This objective can be realized through the application of various ablation techniques. Currently, point-by-point radiofrequency (RF) ablation and cryoballoon (CB) ablation techniques remain widely employed in clinical practice.

Previously, the two methods were extensively compared in a multicenter randomized FIRE AND ICE trial. The trial showed that, at the time, the CB and RF methods where equally effective and safe alternatives in drug-refractory pAF [4]. However, the adoption of new catheter technology was not uniform across the trial; specifically, the second-generation cryoballoon was utilized in 75.6% of patients in the CB arm, while contact force-measuring RF catheters were employed in only 24.7% of patients in the RF group [4]. Moreover, the ablation index formula, which integrates CF, RF time, and RF power within a real-time, non-linear framework, has become available. This sophisticated integration holds promise for optimizing efficacy outcomes in AF ablation procedures [5].

The objective of this prospective, single-center observational trial was to assess and compare the efficacy, safety, and long-term success rates between second-generation CB PVI and AI-guided point-by-point RF ablations in patients undergoing AF ablation for pAF.

## 2. Methods

### 2.1. Study Population

In our single-center trial, we enrolled 154 consecutive patients experiencing symptomatic pAF refractory to AADs. These patients were scheduled for PVI between January 2021 and January 2023 at our university center. Prior to the procedure, participants underwent cardiac computed tomography (CT) scans to evaluate left atrial anatomy, screen coronary status, and rule out left atrial appendage thrombus formation. The left atrium and PVs were segmented, and the resulting 3D reconstruction images were exported to a real-time mapping system (CARTO 3, Biosense Webster, Diamond Bar, CA, USA). The segmentation of all cardiac CT scans led to the categorization of participants into either CB or AI-guided RF groups based on the operators’ decision, influenced by left atrial anatomy. Specifically, cases involving a left common ostium, accessory pulmonary veins, extensive pulmonary vein ostia (>28 mm in diameter), or other anatomical variations that made the use of a single-shot device challenging were assigned to the AI-RF group, while CB ablation was performed in all other cases.

Patients referred for a second ablation, those under the age of 18, or those who declined participation were excluded from this study. The research adhered to the principles outlined in the Declaration of Helsinki and received approval from the regional ethics committee. All participating patients provided written informed consent before being enrolled in this study.

### 2.2. Procedural Workflow

During the procedures, conscious sedation was induced employing fractionated boluses of fentanyl and midazolam while ensuring the preservation of spontaneous breathing and continuous monitoring of oxygen saturation. Following local anesthesia and guided by vascular ultrasound, a decapolar steerable catheter (Dynamic Deca, Bard Electrophysiology, Lowell, MA, USA) was inserted into the coronary sinus (CS) through femoral venous puncture. Trans-septal puncture was performed with intracardiac echocardiography (ICE) guidance via SL0 (Abbott Laboratories, Chicago, IL, USA). Following the initial trans-septal puncture, intravenous unfractionated heparin was promptly administered, and an activated clotting time of >300 s was maintained throughout the entire procedure.

In the AI-RF group, a steerable sheath (Agilis™ NxT, Abbott Laboratories, Chicago, IL, USA) was introduced into the left atrium alongside the SL0 (Abbott Laboratories, Chicago, IL, USA), utilizing either a sliding technique or, in cases where sliding was unsuccessful, a double trans-septal puncture was performed. Then, a contact force-sensing RF ablation catheter (Navistar Thermocool SmartTouch ST NAV, Biosense Webster Inc., Diamond Bar, CA, USA) and a multipolar, steerable circular mapping catheter (Lasso NAV, Biosense Webster Inc., Diamond Bar, CA, USA) were inserted into the left atrium. Anatomical mapping of the left atrium was conducted using the Lasso NAV catheter supported by the CARTO electroanatomical mapping system (Biosense Webster Inc., Diamond Bar, CA, USA). The ablation catheter was configured in a power-controlled mode with a maximum power of 45 W for the anterior wall and 35 W for the posterior wall, employing a maximum temperature of 43 °C. Throughout the RF ablations, the CARTO VISITAG™ Module (Biosense Webster Inc., Diamond Bar, CA, USA) was utilized, with a minimum stability time of 4 s and a maximum location stability range of 2.5 mm. The Visitag Surpoint (i.e., ablation index) was applied, with targets set at 350 for the posterior wall and 450 for the anterior wall. The target interlesion distance was maintained below 5 mm. A point-by-point ablation technique was employed, with real-time monitoring of CF and impedance. CF was maintained within the range of 5 to 15 g during ablation.

In CB group, SL0 was changed over the wire to a 12 Fr steerable sheath (FlexCath Advance, Medtronic, Minneapolis, MN, USA), and a second-generation CB (Arctic Front Advance, Medtronic, Minneapolis, MN, USA) was introduced into the left atrium. A circular mapping catheter (Achieve Mapping Catheter, Medtronic, Minneapolis, MN, USA) equipped with the CB was placed in each PV ostium. Contrast injection was utilized to confirm occlusion. The freezing strategy involved durations of 180–240 s with a minimum temperature set at −60 °C. While freezing the right PVs, periodic fluoroscopy was employed to monitor diaphragm movement, serving as an assessment of the integrity of the phrenic nerve.

The procedural endpoint for the ablation was considered achieved when isolation of all PV was confirmed. The procedure time was defined as the duration from the first femoral vein puncture to the removal of the catheters. Fluoroscopy time and radiation dose were automatically recorded by the fluoroscopy system. The occurrence of major complications, such as vascular complications, pericardial effusion, cardiac tamponade, stroke, or atrio-esophageal fistula, was systematically assessed throughout the entire hospitalization and a 30-day periprocedural period. Postprocedural complications were monitored through physical examination the day after the procedure, with a specific focus on vascular complications at the puncture site. Auscultation of the femoral region was performed, and if a new systolic murmur was detected, duplex ultrasound examination was conducted to assess vascular abnormalities. Additionally, postprocedural complications were documented based on hospital readmissions within 30 days for any ablation-related adverse events.

### 2.3. Follow-Up Period

Following the initial ablation procedure, teleambulatory visits were scheduled at 3, 6, and 12 months, or in the event of recurrent symptoms. The use of AADs was discontinued during the first visit. At each teleambulatory visit, a comprehensive medical history was documented, and arrhythmia symptoms were assessed through telephone interviews. Symptomatic patients were instructed to submit monthly transtelephonic electrocardiogram (ECG) recordings throughout this study. Additionally, they were encouraged to transmit ECGs whenever they experienced symptoms indicative of arrhythmia. We defined recurrences as any atrial arrhythmia lasting more than 30 s, as documented by surface/transtelephonic ECG or smart device recordings.

### 2.4. Statistical Analysis

Data were assessed for normal distribution using the Kolmogorov–Smirnov goodness-of-fit test. Continuous data were expressed either as the mean ± standard deviation (SD) or as the median (interquartile range, IQR), depending on the distribution. Categorical variables were presented as absolute numbers and percentages. For comparisons, the chi-square test, *t*-test, and Mann–Whitney U test were employed as appropriate. A *p*-value < 0.05 was considered statistically significant for all analyses. The statistical analyses were conducted using SPSS 28 software (SPSS, Inc., Chicago, IL, USA).

## 3. Results

A total of 154 patients were included in this study, with 51 assigned to the CB group and 103 to the AI-RF group.

In the demographic analysis, no significant differences were observed between the AI-RF and CB groups across key baseline characteristics (Table 1). The proportion of male patients was similar between the AI-RF group (65.0%) and the CB group (62.7%) (*p* = 0.77). The mean age was also comparable, with 62.2 years in the AI-RF group and 61.1 years in the CB group (*p* = 0.67). Comorbidities such as hypertension and diabetes mellitus were similarly distributed between groups, with hypertension present in 74.8% of the AI-RF group and 80.4% of the CB group (*p* = 0.35) and diabetes mellitus in 17.4% and 16.0%, respectively (*p* = 0.92). Additionally, prior stroke or transient ischemic attack (TIA) was reported in 5.8% of the AI-RF group and 11.8% of the CB group (*p* = 0.12). Regarding heart failure, 11.7% of the AI-RF group and 3.9% of the CB group had this diagnosis, though this difference did not reach statistical significance (*p* = 0.11). Similarly, coronary artery disease and chronic kidney disease were observed at comparable rates between the two groups (*p* = 0.26 and *p* = 0.21, respectively).

Antiarrhythmic drug usage was consistent between the groups, with no antiarrhythmic drug use in 42.7% of the AI-RF group and 49.0% of the CB group (*p* = 0.45). The use of specific antiarrhythmic drugs, such as propafenone, sotalol, and amiodarone, also showed no significant differences between the groups (*p*-values of 0.12, 0.29, and 0.86, respectively).

The procedural and outcome data demonstrated notable differences between the CB and AI-RF groups (Table 2). The CB group had a significantly shorter total procedure time compared to the AI-RF group, with a median of 64 min (IQR 57–74.8) versus 92 min (IQR 76–119, *p* < 0.001). However, the CB group also experienced significantly higher fluoroscopy time and dose, with a median fluoroscopy time of 559 s (IQR 395–868) compared to 167 s (IQR 126–224) in the AI-RF group (*p* < 0.001) and a median fluoroscopy dose of 21.8 mGy (IQR 11.7–40.1) versus 7.65 mGy (IQR 5.21–14.5, *p* < 0.001).

**Figure 1:**
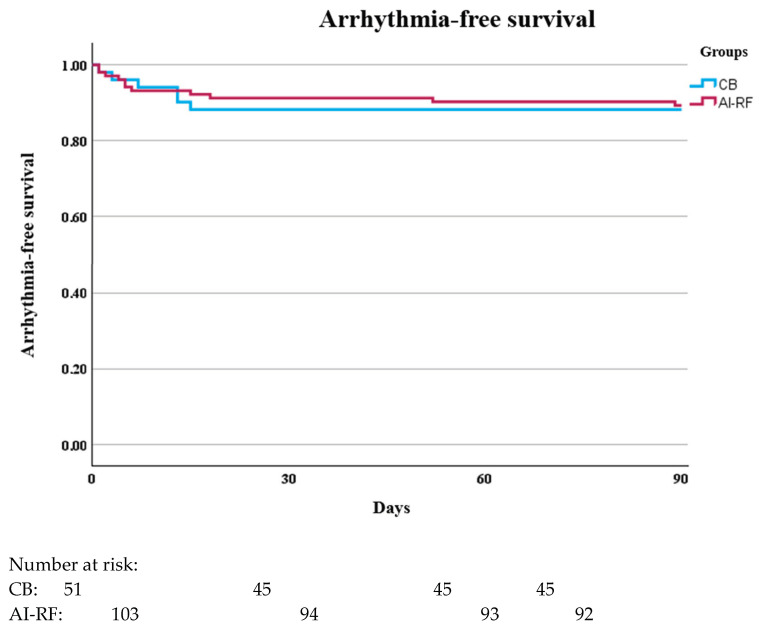
Comparison of Arrhythmia-Free Survival Between CB and AI-RF Groups during the blanking period.

Recurrence rates during the blanking period were similar between the groups (Table 2, Figure 1), with 11.7% in the CB group and 10.7% in the AI-RF group (*p* = 0.84).

**Figure 2:**
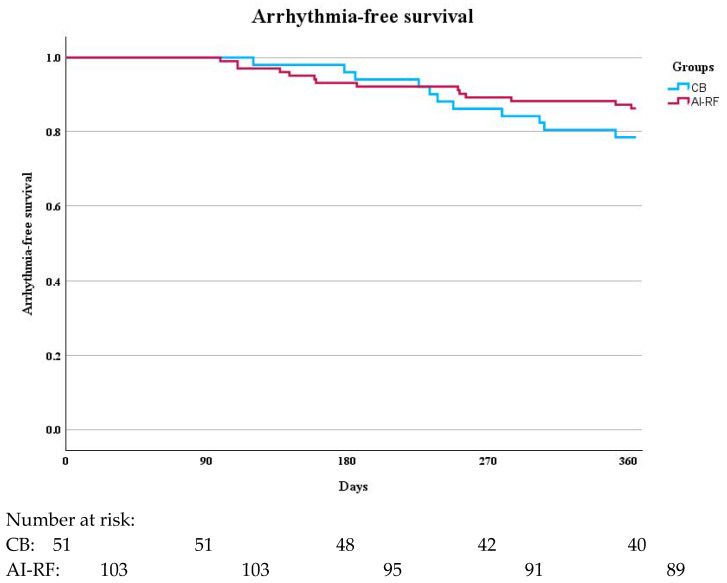
Comparison of Arrhythmia-Free Survival Between CB and AI-RF Groups During the 360-Day Follow-Up Period.

In the 12-month follow-up period, recurrence of AF was observed in 21.6% of patients in the CB group and 13.4% of patients in the AI-RF group (*p* = 0.21, Table 2, Figure 2). The estimated hazard ratio (HR) for arrhythmia recurrence in the CB group compared to the AI-RF group was 1.73 (95% CI: 0.86–3.47), indicating a trend toward higher recurrence in the CB group; however, this difference did not reach statistical significance.

## 4. Discussion

In our single-center, prospective study, we analyzed the procedural outcomes and recurrence rates during a 12-month follow-up period in patients with pAF undergoing PVI via either AI-guided point-by-point RF or CB ablation. We found shorter procedure time and longer fluoroscopy time associated with CB ablations and similar rates of recurrence during blanking and follow-up.

Catheter ablation stands as the foremost globally practiced intervention for addressing AF, with its fundamental principle centered on the electrical isolation of the PVs. These isolation of the PVs could be achievable with different forms of energy (i.e., thermal or non-thermal) and approaches including single-shot and point-by-point ablation procedures.

The first human trial data on single-shot cryoballoon ablations for AF were published in 2007. These initial results demonstrated a short learning curve and established that cryoballoon ablation is non-inferior to open irrigated RF technology in terms of arrhythmia-free outcomes [6,7]. The initial generation of CB catheters underwent subsequent refinement, leading to the development of second-generation CB catheters. These advancements facilitated a more uniform cooling effect over a broader catheter surface area, thereby enhancing the procedural outcomes of CB ablation procedures [8].

The FIRE and ICE trial was the first prospective, multicenter randomized study comparing CB to point-by-point RF PVI procedures. During the study, the procedural data and long-term success rates of 762 patients were analyzed in a 1.5-year-long follow-up period. According to the study results, the CB method proved to be non-inferior in terms of recurrence rate (CB: 34.6%; RF: 35.9%; *p* < 0.001 for non-inferiority). There were no significant differences in complication rates. Using the CB method resulted in a significantly shorter total procedural time but showed longer total fluoroscopy time [4].

Throughout the trial period, advancements in cryoballoon and RF ablation catheters were introduced into the commercial market and incorporated into the study at the discretion of the investigators. However, there was an unequal adoption of new catheter technology within the trial, with second-generation CB utilized in 75.6% of patients in the CB group, while advanced-generation, contact force-sensing RF catheters were employed in only 24.7% of patients in the RF group [4,5,6,7,8,9].

The introduction of CF-sensing RF catheters has significantly enhanced procedural parameters and success rates in PVI ablations by allowing operators to monitor applied lesions. A meta-analysis comprising 22 studies with 4094 patients demonstrated that compared to conventional non-CF catheters, CF utilization led to significantly reduced total procedure time, fluoroscopy time, and ablation time in AF ablation procedures. Additionally, in patients with pAF, there was a notable improvement in long-term success rates [10].

In addition to the introduction of CF-sensing catheters, the incorporation of the ablation index into clinical practice was facilitated by the experimental research conducted by Nakagawa et al. [11]. This composite metric, which integrates parameters such as CF, power, and duration within a weighted algorithm, demonstrated a high level of accuracy in predicting lesion depth within the canine ventricle.

Moreover, the CLOSE protocol was published in 2018. This protocol represents an innovative strategy designed to create contiguous and optimized RF lesions around the pulmonary veins (PVs), with a focus on achieving an interlesion distance of less than or equal to 6 mm and an ablation index of 400 at the posterior wall and 550 at the anterior wall [12,13]. A single-center study revealed that CLOSE protocol-guided PVI demonstrated a higher rate of first-pass isolation and reduced procedure duration when compared to the standard CF-guided approach. Moreover, during the 12-month follow up, the freedom of atrial arrhythmias following a single procedure was comparatively lower with CLOSE-guided PVIs [14].

High-power short-duration radiofrequency ablation (HPSD) has gained increasing attention for its potential to enhance procedural efficiency while minimizing collateral tissue damage. Kumar et al. demonstrated that HPSD settings can improve lesion formation, reduce total ablation time, and potentially lower the risk of complications such as steam pops and cardiac tamponade compared to low-power long-duration (LPLD) ablation strategies. This is particularly relevant given the anatomical complexity of the thick ridge between the left atrium and the upper left pulmonary veins, where achieving effective lesion formation while minimizing procedural time remains a critical challenge. The optimized energy delivery of HPSD ablation may help overcome these challenges by providing more efficient and controlled lesion formation [15]. Additionally, Kotadia et al. highlighted that HPSD strategies may facilitate more efficient tissue heating, leading to a reduction in the number of lesions required for pulmonary vein isolation while ensuring superior lesion durability and continuity [16]. Furthermore, La Fazia et al. demonstrated that HPSD ablation using 50 W for 10–15 s resulted in a higher rate of transmural lesion formation during posterior wall ablation in PVI compared to other power settings, including 40 W for 10–15 s and 90 W for 4 s [17].

To date, there are limited comparative data available on ablation approaches involving AI-RF and second-generation CB for PVIs. Previously, a randomized single-center trial investigated these two approaches. In this study, 150 patients undergoing de novo PVI for pAF were randomized. The recurrence rate was higher in the CB group during the 3-month blanking period (18.67% vs. 8%). However, there was no significant difference between the groups in terms of 12-month recurrences without antiarrhythmic drugs (14.7% vs. 13.3%). Surprisingly, no significant disparity was observed concerning fluoroscopy time (CB: 8.56 ± 3.18 min; AI-RF: 9.66 ± 3.86 min; *p* = 0.06) or dose area product (CB: 390 ± 268.57 cGy/cm^2^; AI-RF: 330.84 ± 150.36 cGy/cm^2^; *p* = 0.1) during the procedures [18].

Contrary to the study results, we found no difference in recurrence rates between the two groups during the blanking period. In our protocol, antiarrhythmic drugs were not discontinued preprocedurally or immediately after the procedure but only during the 3-month follow-up visit. This may explain the lack of difference in recurrent PVI rates observed during the blanking period. Additionally, while the CB group had a significantly shorter procedural time in our study, fluoroscopy exposure was notably lower in the AI-RF group.

The relationship between left atrial fibrosis and PVI outcomes has been a subject of significant research interest. Fibrosis is a structural remodeling process that not only contributes to the initiation and maintenance of AF but also influences the success rates of ablation techniques aimed at achieving long-term sinus rhythm. Higher degrees of fibrosis have been associated with increased recurrence of arrhythmias following ablation [19]. Karakasis et al. categorize fibrosis into two distinct types: reactive fibrosis, which occurs as a response to injury or stress, and replacement fibrosis, which results from the loss and subsequent substitution of cardiomyocytes with fibrous tissue. The relative contribution of these fibrosis subtypes varies among individuals and is influenced by factors such as age, sex, and comorbidities (e.g., hypertension, heart failure), making standardized ablation strategies challenging. This underscores the role of fibrosis as a critical substrate for AF persistence and highlights its impact on procedural success [20]. Both cryothermal and radiofrequency energy interact differently with atrial tissue, particularly in fibrotic regions. Åkerström et al. demonstrated that CB ablation generates less thrombogenic lesions and creates a more homogeneous lesion profile, which may be advantageous when ablating fibrotic tissue. The uniform cooling effect of cryothermal energy facilitates durable and consistent PVI [21]. In contrast, RF ablation induces tissue necrosis followed by an inflammatory response, ultimately leading to fibrotic scar formation. However, lesion quality can vary significantly depending on local tissue characteristics, including pre-existing fibrosis, which may impede effective ablation and increase the risk of conduction recovery across previously isolated veins. Furthermore, the inflammatory process triggered by RF ablation may complicate tissue healing and contribute to arrhythmia recurrence [22]. The variability in lesion formation when ablating fibrotic substrates underscores the importance of optimized ablation parameters, particularly with the use of ablation index, which integrates contact force, power, and duration to enhance procedural efficacy in fibrotic regions [23].

The advantages of the cryoballoon technique, as a single-shot approach, include a shorter learning curve and typically faster procedure times compared to the longer learning curve and procedure duration associated with point-by-point radiofrequency PVI [24]. Due to the inherent nature of these techniques, CB ablations involve higher radiation exposure. However, the availability of visualizable steerable sheaths in recent years has allowed for a reduction in radiation exposure during procedures, making fluoroscopy-free catheter ablation achievable [25,26]. The zero-fluoroscopy PVI technique is effective and safe [27], even for operators without prior experience [28]. The use of ICE guidance has also been shown to facilitate zero-fluoroscopy CB ablation. A study published in 2023 reported that ICE-guided CB ablation resulted in procedural and ablation times comparable to the conventional approach, with similar recurrence rates, while significantly reducing radiation exposure [29]. Even in cases where complete zero-fluoroscopy was not achieved, the incorporation of ICE into the PVI workflow led to a substantial reduction in radiation exposure [30].

With CB and AI-RF techniques each offering distinct procedural benefits, our findings emphasize the importance of individualizing treatment strategies for pAF. As advancements in ablation technology evolve, further research could help to streamline these approaches, reduce fluoroscopy exposure, and enhance long-term success in AF management.

## 5. Limitations

Several limitations should be noted. First, as a single-center study, the generalizability of our findings is limited. Additionally, the non-randomized design may introduce unaccounted variables that could have impacted the results. Moreover, the selection of the ablation technique based on CT findings resulted in significant differences in left atrial anatomy between the groups. This study was underpowered to detect a statistically significant difference in recurrence rates. Future studies with larger sample sizes are warranted to determine whether there are any differences in long-term success rates between the two techniques.

## 6. Conclusions

CB and AI-RF PVIs demonstrated equal effectiveness and safety. However, CB ablations were associated with shorter procedure times, though they required higher fluoroscopy exposure compared to point-by-point RF ablations guided by the ablation index.

## Figures and Tables

**Table 1:** Baseline characteristics. Abbreviation: TIA—transient ischemic attack.

	Group AI-RF (*n* = 103)	Group CB (*n* = 51)	*p*-Value
Male, *n* (%)	67 (65.0)	32 (62.7)	0.77
Age, y	62.2 (49.6; 70.0)	61.1 (51.6; 69.0)	0.67
Hypertension, *n* (%)	77 (74.8)	41 (80.4)	0.35
Diabetes mellitus, *n* (%)	18 (17.4)	9 (16.0)	0.92
Prior stroke/TIA, *n* (%)	6 (5.8)	5 (11.8)	0.12
Heart failure, *n* (%)	12 (11.7)	2 (3.9)	0.11
Coronary artery disease, *n* (%)	17 (16.5)	5 (7.7)	0.26
Chronic kidney disease, *n* (%)	15 (15.0)	4 (7.8)	0.21
No antiarrhythmic drug during the blanking period, *n* (%)	44 (42.7)	25 (49.0)	0.45
Propafenone during the blanking period, *n* (%)	34 (33.0)	14 (27.5)	0.12
Sotalol during the blanking period, *n* (%)	3 (2.9)	3 (5.9)	0.29
Amiodarone during the blanking period, *n* (%)	19 (18.4)	8 (15.6)	0.86

**Table 2:** Our results are summarized in this table. For total procedure time, total fluoroscopy time, and fluoroscopy dose, median values and interquartile values are shown.

	CB (*n* = 51)	AI-RF (*n* = 103)	*p*-Value
Total procedure time, min	64 (57;74.8)	92 (76; 119)	<0.001
Total fluoroscopy time, s	559 (395; 868)	167 (126; 224)	<0.001
Total fluoroscopy dose, mGy	21.8 (11.7; 40.1)	7.65 (5.21; 14.5)	<0.001
Recurrence rate during the blanking period, %	11.7	10.7	0.84
Recurrence rate outside the blanking period, %	21.6	13.4	0.21

## Data Availability

The original contributions presented in this study are included in the article. Further inquiries can be directed to the corresponding author.

## Abbreviations

AAD	antiarrhythmic drug
AF	atrial fibrillation
AI-RF	ablation index-guided radiofrequency ablation
CB	cryoballoon
CT	computed tomography
CS	coronary sinus
ECG	electrocardiogram
HPSD	high-power short-duration radiofrequency ablation
ICE	intracardiac echocardiography
IQR	interquartile range
LPLD	low-power long-duration radiofrequency ablation
PV	pulmonary vein
PVI	pulmonary vein isolation
pAF	paroxysmal atrial fibrillation
SD	standard deviation
TIA	transient ischemic attack
